# Long-term survival trends of gastric cancer patients between 1972 and 2011 in Qidong

**DOI:** 10.1186/s40880-015-0058-y

**Published:** 2015-10-19

**Authors:** Yong-Sheng Chen, Jian-Guo Chen, Jian Zhu, Yong-Hui Zhang, Lu-Lu Ding

**Affiliations:** Qidong Liver Cancer Institute, Qidong People’s Hospital, Qidong, Jiangsu 226200 P.R. China; Department of Epidemiology, Nantong University Tumor Hospital/Institute, Nantong, Jiangsu 226361 P.R. China

**Keywords:** Gastric neoplasms, Cancer registration, Survival, Trends, Qidong

## Abstract

**Background:**

There have been few reports on long-term survival of gastric cancer patients. This study analyzed the survival data of gastric cancer patients obtained from the population-based Qidong Cancer Registry between 1972 and 2011, providing a basis for evaluation of gastric cancer treatment and prognosis.

**Methods:**

The cumulative observed survival rate and relative survival rate of gastric cancer patients were calculated using Hakulinen’s method via the SURV3.01 software, which was developed by the Finnish Cancer Registry. The date of the last follow-up for the survival status of the 15,401 registered cases was April 30, 2012.

**Results:**

The 1-, 5-, 10-, 20-, and 30-year observed survival rates were 33.82%, 14.18%, 10.35%, 6.63%, and 4.19%, respectively, and the 1-, 5-, 10-, 20-, and 30-year relative survival rates were 35.43%, 18.13%, 17.50%, 21.96%, and 32.84%, respectively. For males, the corresponding observed survival rates were 34.50%, 14.40%, 10.42%, 6.46%, and 4.05%, and the corresponding relative survival rates were 36.23%, 18.67%, 18.28%, 23.73%, and 38.61%. For females, the corresponding observed survival rates were 32.62%, 13.80%, 10.22%, 6.95%, and 4.46%, and the corresponding relative survival rates were 34.03%, 17.21%, 16.28%, 19.70%, and 26.78%. Significant differences in relative survival rates were observed between sexes (*P* = 0.003). For the 15–34, 35–44, 45–54, 55–64, 65–74, and 75+ age groups, the 5-year relative survival rates were 16.13%, 21.77%, 18.63%, 12.61%, 7.99%, and 2.94%, respectively, and the 10-year relative survival rates were 16.49%, 22.83%, 20.50%, 15.97%, 15.88%, and 15.73%, respectively. Remarkable improvement could be observed for the 5-, 10-, and 15-year relative survival rates in Qidong beginning in the 1980s.

**Conclusion:**

The survival outcome of registered gastric cancer cases in Qidong showed gradual progress over the past two decades.

## Background

Gastric cancer is the fifth most common malignancy in the world according to statistics from GLOBOCAN 2012, with nearly one million new cases per year (952,000 cases, 6.8% of the total) [1]. More than 70% of cases (677,000 cases, with 456,000 in males and 221,000 in females) occur in developing countries, and half of the world total occurs in East Asia (mainly in China).

In the Western Pacific Region, the stomach is the second leading cancer site in males and the fourth most common cancer site in females [2]. In mainland China, gastric cancer is the third most common malignant disease, which makes it a main disease burden among Chinese residents. In 2009, the age-standardized rate (ASR) of incidence adjusted by Chinese population (ASRIC) for gastric cancer was 17.85 per 100,000 (25.37 per 100,000 in males and 10.62 per 100,000 in females) [3]. The ASR of incidence adjusted by world population (ASRIW) was 22.7 per 100,000 (32.8 per 100,000 in males and 13.1 per 100,000 in females) [1]. In Qidong, gastric cancer has been the second most common cancer over the past 40 years, with a crude incidence of 34.26 per 100,000 and an ASRIW of 25.59 per 100,000, accounting for 16.60% of all cancer cases. The annual percentage change (APC) in the ASRIW has been −2.06%, with an accelerating decline between 1972 and 2011 [4].

To date, gastric cancer is usually detected late, hence patient survival rates are poor in most settings [5]. Reports on long-term survival of gastric cancer patients are rare. The prognosis of neoplasms has been shown to be poor in China [6], so the 1-year survival rate is used to reflect patients’ short-term living status, whereas the 5-year survival rate is generally used as an indicator of patients’ long-term living status. The relative survival rate calculated from population-based cancer registry data, which is based on the concept of the probability of survival at the same age, with the same sex, and in the same period, is a helpful metric to compare the prognoses of patients from different areas [7, 8].

Recently, to develop effective public health policies for cancer control, China has made efforts to improve its systematic recording of cancer data and to report the survival rates for different cancers, including gastric cancer, adjusted by age, sex, and locality [6]. However, these efforts provide only a very short interval (2003–2005) for evaluating the survival of patients with gastric cancer in only limited areas. To better understand the effects of changes or improvement in the outcomes of gastric cancer diagnosis and treatment in a sentinel rural population and to provide valuable insights for future planning of preventative activities by health authorities, this study analyzed the survival rates of gastric cancer patients from a population-based cancer registry in Qidong over the period of 1972–2011. Qidong is located at the mouth of the Yangtze River, to the north of Shanghai. Qidong is a city (former county) in Jiangsu Province, covering an area of 1234 km^2^ and containing a registered resident population of 1.12 million as of the end of 2013.

The Qidong Cancer Registry was designated as a cancer registration repository by the local health authority in 1972, with compulsory reporting by health care workers. Several years later, cancer registration was mandated to be compulsory by the provincial health authority. This cancer registry is now a member of the national monitoring program (known as the National Cancer Registration Network) [9, 10] of the National Central Cancer Registries of China, supported by the Ministry of Finance and the Ministry of Health of China: Tumor Follow-up Registration Programs (MF2008-293, 2009-193, and 2010-90).

## Methods

### Data collection

The Qidong Cancer Registry uses both active and passive methods for cancer data collection. As a member of the International Association of Cancer Registries, the Qidong Cancer Registry implemented the association’s standards for quality, completeness, timeliness, and unresolved duplicate records. The International Classification of Diseases, 10th revision (ICD-10), is used for cancer classification; gastric cancer is coded as C16. All data files received from lower-level registries and all other hospitals are compared with cancer report lists and death certification notifications (DCNs) to track down missing cases and to exclude duplicate registrations. When the registry personnel receive the death notification first, the patient’s medical records are reviewed, or a home visit is carried out to obtain further information. Those patients without any death information would be presumed to be still alive (“survivors”), and active follow-up is conducted as a routine procedure [9]. In addition, periodic follow-up is performed every 5 years. The deadline for the latest follow-up for the data set studied here was April 30, 2012.

### Quality control of data

The percentage of morphologic verification (MV%), the ratio of mortality to incidence (M/I), and the percentage of death certificate only cases (DCO%) were used as indices of quality control. The DCN data from the Qidong All-Death-Cause Registration System [9] were linked to and compared with new registered cases if the patient had died. The survival of each case was determined as the duration from the date of initial diagnosis to the date of death due to gastric cancer, the date of death due to other diseases, the date of last follow-up, or the date of closing for those who were still alive. The Qidong Cancer Registry data had been included in “Cancer Incidence in Five Continents” (CI5) [1, 11, 12] and other publications [9, 10]. The reliability of these data had earned recognition in both domestic and international studies [9–12].

### Statistical methods

The cumulative observed survival rate and relative survival rate were computed for six age groups (15–34, 35–44, 45–54, 55–64, 65–74, and 75+) and nine calendar periods (1972, 1973–1977, 1978–1982, 1983–1987, 1988–1992, 1993–1997, 1998–2002, 2003–2007, and 2008–2011). The observed survival and relative survival rates were calculated by using the SURV3.01 software developed by Hakulinen from the Finnish Cancer Registry [13]. The relative survival rate was calculated by dividing the observed survival rate by the expected survival rate for a group of people in the general population who are similar to the patient group with respect to sex, age, and the calendar period of observation [14], i.e., *S*_*c*_*(t)* = *S*_*o*_*(t)*/*S*_*e*_*(t)*, where *S*_*c*_*(t)* is the relative survival rate, *S*_*o*_*(t)* is the observed survival rate, and *S*_*e*_*(t)* is the expected survival rate, and *S*_*e*_*(t)* = *∑n*_*x*_*S*_*ex*_*(t)*/*∑n*_*x*_, where *n*_*x*_ is the number of patients being followed up at the age of *x* and *S*_*ex*_*(t)* is the survival rate at the time point *t* at the age of *x*. The expected survival rate for a group of people in the general population who are similar to the patient group with respect to sex, age, and the registered calendar year of observation was calculated using the Qidong life tables [7, 15] for the years 1974–2011.

## Results

### Quality-control indices

There were 15,401 cases of gastric cancer from 1972 to 2011, among which 9804 were males and 5597 were females (the ratio of males to females was 1.75:1). The MV% was 55.52%, the M/I ratio was 89.49, and the DCO% was 0.19%. The number of deaths due to gastric cancer was 13,675 (88.79%), and the number of deaths due to other diseases was four (0.03%). A total of 452 (2.93%) patients were lost to follow-up, and 1270 (8.25%) were still alive by the end of 2011.

### Observed survival and relative survival of gastric cancer patients

**Fig. 1:**
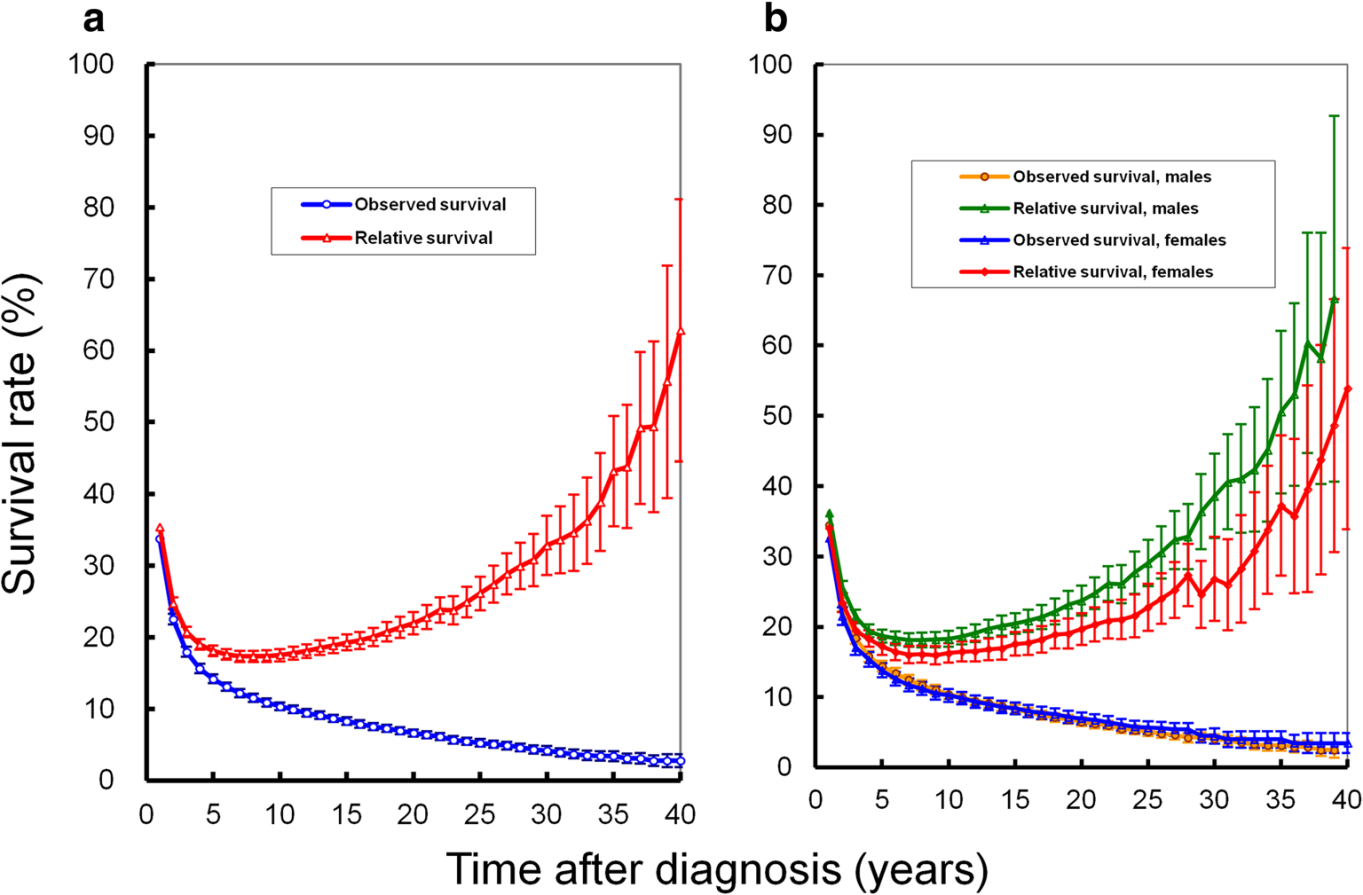
Trends in observed survival rate and relative survival rate of gastric cancer patients in Qidong, China between 1972 and 2011. **a** Observed survival and relative survival rates of all patients. **b** Observed survival and relative survival rates for males and females

The 1-, 3-, 5-, 10-, 15-, 20-, and 30-year observed survival rates were 33.82%, 17.97%, 14.18%, 10.35%, 8.29%, 6.63%, and 4.19%, respectively, and the 1-, 3-, 5-, 10-, 15-, 20-, and 30-year relative survival rates were 35.43%, 20.74%, 18.13%, 17.50%, 19.27%, 21.96%, and 32.84%, respectively. The observed survival and relative survival rates by year and their standard errors (SEs) are depicted in Fig. 1a.

### Survival rate by sex

The 1-, 3-, 5-, 10-, 15-, 20-, and 30-year observed survival rates were 34.50%, 18.46%, 14.40%, 10.42%, 8.24%, 6.46%, and 4.05%, respectively, for male patients, and were 32.62%, 17.11%, 13.80%, 10.22%, 8.40%, 6.95%, and 4.46 %, respectively, for female patients, with no significant differences between males and females. The 1-, 3-, 5-, 10-, 15-, 20-, and 30-year relative survival rates were 36.23%, 21.48%, 18.67%, 18.28%, 20.47%, 23.73%, and 38.61%, respectively, for male patients, and were 34.03%, 19.47%, 17.21%, 16.28%, 17.56%, 19.70%, and 26.78%, respectively, for female patients, with significant differences between males and females (χ^2^ = 24.79, *P* = 0.003). The results of the observed survival and relative survival rates by sex are shown in Fig. 1b.

### Survival rate by age

Significant differences in relative survival rates were found among the age groups of 15 years and over both for males (*χ*^2^ = 440.72, *P* < 0.001) and for females (*χ*^2^ = 426.16, *P* < 0.001). The highest 5-year observed survival and relative survival rates were seen in the age group of 35–44, and the highest 10-year observed survival and relative survival rates were seen in the age groups of 45–54 and 35–44, respectively. The patients in the age group of 75+ experienced the worst prognosis. For males, the highest 5- and 10-year observed survival rates were seen in the age group of 35–44; the highest 5- and 10-year relative survival rates were seen in the age group of 15–34. For females, the highest 5-year observed survival rate was seen in the age group of 35–44, but the highest 10-year observed survival rate was seen in the age group of 45–54; the highest 5- and 10-year relative survival rates were seen in the age group of 35–44 (Table 1).

**Table 1:** The 5- and 10-year observed survival and relative survival rates for gastric cancer patients in different age groups in Qidong, China between 1972 and 2011

Age group	Observed survival rate (%)						Relative survival rate (%)					
	5-year			10-year			5-year			10-year		
	Males	Females	Total	Males	Females	Total	Males	Females	Total	Males	Females	Total
15–34	20.05	21.82	15.89	20.30	22.01	14.09	23.60	16.42	16.13	23.71	16.94	16.49
35–44	29.12	25.59	18.61	29.92	16.12	19.17	21.32	24.43	21.77	21.58	26.01	22.83
45–54	23.37	22.94	18.13	24.51	23.87	19.22	22.12	18.91	18.63	22.67	21.21	20.50
55–64	17.28	17.25	13.59	19.24	18.89	15.71	17.17	12.18	12.61	18.14	16.09	15.97
65–74	10.89	11.90	9.31	14.36	15.14	15.63	14.11	7.38	7.99	16.68	16.08	15.88
75+	6.32	5.81	2.86	14.80	12.27	11.99	5.21	3.05	2.94	9.90	21.52	15.73
Total	14.40	14.18	10.22	18.67	18.13	16.28	13.80	10.42	10.35	17.21	18.21	17.50

### Survival rate by period

Table 2 shows that the relative survival rates of gastric cancer patients gradually increased over the past 40 years. The 1-year relative survival rate was 34.24% in 1972 and 45.15% in 2008–2011. The 5-year relative survival rate was 14.24% in 1972 and 26.67% in 2003–2007. The 10-year relative survival rate increased from 11.10% in 1972 to 17.99% in 1998–2002.

**Table 2:** Relative survival rate of gastric cancer patients by periods in Qidong, China between 1972 and 2011

Period	Relative survival rate (%)						
	1-year	3-year	5-year	10-year	15-year	20-year	30-year
1972	34.24	16.77	14.24	11.10	9.55	5.63	7.95
1973–1977	33.71	14.89	11.79	9.10	8.88	9.10	15.49
1978–1982	31.75	16.92	13.53	11.91	11.80	12.55	15.07
1983–1987	32.07	17.89	14.47	13.12	15.04	18.64	NA
1988–1992	32.85	20.25	18.09	18.91	21.90	25.31	NA
1993–1997	30.57	18.43	16.92	17.73	20.25	NA	NA
1998–2002	35.66	20.20	18.01	17.99	NA	NA	NA
2003–2007	42.00	28.26	26.67	NA	NA	NA	NA
2008–2011	45.15	31.08	NA	NA	NA	NA	NA

*NA* not available

## Discussion

Our results show an increasing trend in the survival rate of gastric cancer patients in Qidong over the past 40 years, which may be a reflection of improvements in cancer care services and cancer prevention.

The observed survival rate generally decreases over time within a group of patients, but the relative survival rate does not always do so. Our data show that the 1–9-year relative survival rates decreased with increasing survival duration, whereas the 10–40-year relative survival rates increased with survival duration, indicating that the patients with gastric cancer who survived more than 10 years had a lower probability of death due to other causes than the general population did.

Our findings indicate that female gastric cancer patients experienced a worse prognosis than did males. This outcome is similar to survival data from Japan [16] and Korea [17]. Males aged 15–34 and females aged 35–44 experienced the highest relative survival rates, which may reflect disparities between sexes and ages. In Qidong, the proportion of patients with gastric cancer who underwent surgical resection was higher in the younger-age groups than in the older-age groups. Young males have a generally healthy status, which may explain their better outcome than elder males in the present study. However, the reason why middle-age female patients had a significantly better prognosis than females in other age groups is unclear.

The prognosis of gastric cancer in Qidong is not very good compared with that in developed countries [8, 18] and in developed areas of China [19]. However, when the cumulative 40-year data were divided into nine periods using the data from 1972 as a baseline for comparison, we found that the 1-, 5-, 10-, and 15-year relative survival rates were getting better with the calender periods, indicating that the prognosis has been improving with the expansion of health services and the economic development in this area.

At present, there are few available data on long-term survival rates of gastric cancer patients from studies similar to our population-based series (Table 3). In China, the 5-year survival rate of gastric cancer patients in Qidong was lower than the rates in urban areas such as Beijing [19], Shanghai [20], and Tianjin [21] and was close to the rates in rural areas such as Changle, in Fujian Province [22], and Cixian, in Hebei Province [23]. These findings indicate that there is a substantial gap in gastric cancer patient survival between rural and urban areas in China. Survival rates of gastric cancer patients are far better in Japan [16], South Korea [17], and Germany [24] than in China [6]. In the United States, the survival was better in 2003–2009 than in 1975–1977, indicating improvement over the past few decades [18]. Global disparities in gastric cancer patient survival exist. In the recent Concord II study, for patients diagnosed during 2005–2009, the age-standardized 5-year net survival rate of gastric cancer patients was very high in South Korea (58%), Japan (54%), and Mauritius (41%), and the 5-year survival rate varied widely between registries in Africa, Asia, and Central and South America [25]. In Japan, a report showed that the 5-year survival rate of gastric cancer patients reached 62.1% during the 1990s [16], whereas in a recent report from Estonia, the gastric cancer patient survival rate in 1995–2006 was 20% in males and 23% in females [26]. Furthermore, in certain undeveloped countries, such as the African countries Gambia [7] and Uganda [27], the 3-year survival rate of gastric cancer patients was only 8.5%, and the 5-year survival rate was zero.

**Table 3:** Comparison of 5-year survival rate of gastric cancer patients between Qidong and different countries and areas

Area	Sex	Observed survival rate (%)	Relative survival rate (%)	Period
China [6]	Males + females	NA	27.40	2003–2005
	Males	NA	27.90	2003–2005
	Females	NA	26.50	2003–2005
Gambia [7]	Males + females	NA	3.00	1993–1997
Korea [17]	Males	NA	43.80	1993–1997
	Females	NA	43.00	
	Males	NA	50.30	1998–2002
	Females	NA	48.70	
USA [18]	Males	NA	14.40	1975–1977
	Females	NA	16.60	
	Males	NA	26.70	2003–2009
	Females	NA	32.00	
Beijing, China [19]	Males	10.40	NA	1982–1983
	Females	11.30	NA	
	Males	17.60	NA	1987–1988
	Females	19.50	NA	
Shanghai, China [20]	Males + females	25.87	35.24	2002–2006
Tianjin, China [21]	Males	13.00	23.97	1981–1985
	Females	13.00	15.70	
Changle, China [22]	Males	13.37	NA	1989–1998
	Females	5.58	NA	
Cixian, China [23]	Males + females	15.69	17.35	2000–2002
Saarland, Germany [24]	Males + females	NA	35.10	2000–2002
Japan [16]	Males + females	54.40	62.10	1993–1996
Estonia [26]	Males	NA	20.00	1995–2006
	Females	NA	23.00	
Kampala, Uganda [27]	Males + females	NA	0.00	1993–1997

*NA* not available

Survival of gastric cancer patients is influenced by many factors, including the clinical stage at diagnosis, the pathologic subtype of the tumor, the biological behavior of tumor cells, and the degree of tumor cell differentiation. Ideally, these factors should be considered while analyzing and comparing survival rates from different settings or areas. However, these data are not easy to obtain by a population-based cancer registry.

The results from our cancer registry provide a long-term assessment for survival of gastric cancer patients in China and provide a platform for a comprehensive survey of prognostic factors as well as health care services for gastric cancer patients in rural areas.
